# The mediating effect of resilience between physical activity and mental health: a meta-analytic structural equation modeling approach

**DOI:** 10.3389/fpubh.2024.1434624

**Published:** 2024-10-01

**Authors:** Hao Lin, Yuying Zhu, Qingzao Liu, Shan Li

**Affiliations:** ^1^College of Physical Education, Chengdu University, Chengdu, China; ^2^School of Economics and Management, Shanghai University of Sport, Shanghai, China

**Keywords:** physical activity, mental health, resilience, meta-analysis, structural equation modeling

## Abstract

**Background:**

While the correlation between physical activity (PA) and mental health is well known, the mediating mechanism of resilience between the two variables remains unclear.

**Objectives:**

To systematically evaluate the relationship between PA and mental health, and explore the mediating role of resilience between them.

**Methods:**

A systematic search of electronic databases (PubMed, Web of Science, ProQuest, Ebsco, China Knowledge, and China Biomedical Database) was conducted to identify relevant studies, and meta-analytic procedures were used to assess the strength of the relationships between PA and mental health. Furthermore, a meta-analytic structural equation model (MASEM) was used to assess the mediating effects of resilience, ensuring the reliability of our findings.

**Results:**

The findings of 15 studies (17,043 subjects) were subjected to meta-analysis and route analysis. The findings of the meta-analysis revealed a statistically significant positive correlation of 0.288 (95% CI, 0.166–0.402) between PA and positive indicators of mental health, as well as a statistically significant negative correlation (95% CI, −0.342 to −0.171) with negative indicators of mental health. Furthermore, the results of MASEM path analysis indicated that PA may indirectly impact both positive and negative indicators of mental health through the mediating factor of resilience. The indirect effect values were 0.108 (95% CI, 0.080–0.141) and −0.074 (95% CI, −0.100 to −0.051), respectively, accounting for 40.15% of the total effect value and 28.91%.

**Conclusion:**

Physical activity is positively correlated with positive indicators of mental health and negatively correlated with negative indicators of mental health. Moreover, PA can positively influence mental health through the mediating role of resilience.

## 1 Introduction

With the advancement of civilization and the accelerated pace of individuals’ lives, mental health has gradually received societal focus and emerged as a prominent area of research in various fields, including psychology, preventive medicine, and epidemiology. Researchers have proposed a two-factor model of mental health influenced by the principles of positive psychology. This model suggests that mental health is not simply the absence of mental illness but a condition that encompasses the presence of positive indicators, such as life satisfaction, and the absence of negative indicators, such as depression (1–3). Measures of positive indicators of mental health primarily used the Positive Mental Health Scale (PMH-scale) (4), the WHO Happiness Index (5), the Psychological General Well-Being Index (PGWBI) (6), and the Life Satisfaction Scale (7). Negative indicators were measured using the Center for Epidemiologic Studies Depression Scale (CES-D) (8), the Depression Anxiety Stress Scale (DASS-21) (9), the GAD-7 Anxiety Disorder Scale (10), and the SAS-2 Anxiety Questionnaire (11). While there may be variations in the aforementioned measures, the scales themselves encompass the positive and negative signs of mental health, respectively. Therefore, the proposal and application of the two-factor model of mental health lay a solid foundation for a more comprehensive and accurate assessment of an individual’s mental health.

### 1.1 The relationship between PA and mental health

Among the various determinants influencing mental health, physical activity (PA) has become a well-known factor. Intervention studies have demonstrated the effectiveness of PA in treating depression (12, 13) and anxiety (14, 15). Furthermore, observational studies have provided evidence that PA is negatively correlated with negative indicators of mental health such as depression (16, 17), anxiety, and stress (18–20) symptoms. Engaging in PA that involves self-entertainment for psychological gratification may help alleviate adverse emotions and influence human health or subjective well-being (21). Epidemiologic studies indicate that PA is associated with certain indicators of subjective well-being (22) and that engaging in moderate-intensity physical activity positively affects mood (23). Therefore, most studies have shown that PA can directly influence mental health by enhancing individuals’ mental state and reducing negative emotions. However, Hearon et al. (24) found a positive correlation between anxiety sensitivity and PA in adult individuals (*r* = 0.43). Established meta-analyses have mostly explored the impact of PA on mental health with pooled intervention studies (14, 25). Conversely, correlational meta-analyses only considered the relationship between PA and single indicators of mental health (26, 27). The validity and practicality of applying the two-factor model in meta-analysis studies of mental health were verified in the study conducted by Hu et al. (28). Therefore, a meta-analysis of the correlation between PA and mental health using the two-factor model of mental health would allow for more comprehensive results.

### 1.2 Mediating effects of resilience

With the advancement of comprehensive research, current researchers are increasingly focusing on the mediating processes through which PA influences mental health. These mechanisms encompass various variables, including resilience, social support, self-efficacy, and psychological capital. An examination of existing literature indicates that most studies have concentrated on investigating the influence of resilience on mental health. Resilience refers to an individual’s ability or trait to cope with stress, frustration, and trauma. It is a crucial positive psychological quality (29) with an intricate and multidimensional relationship with PA and mental health (30). Researchers found a positive correlation between PA and resilience (31, 32). Individuals’ psychological resilience improves with regular PA (33), while the fulfillment of individual psychological needs further improves resilience during exercise (34). Numerous empirical studies have found that resilience is positively correlated with positive indicators of mental health (35) and negatively correlated with negative indicators of mental health (36, 37). Moreover, resilience can be projected to predict mental health indicators, such as life satisfaction (38), depression (39), and anxiety (40). Empirical studies have confirmed the mediating effect played by resilience between PA and mental health; however, the findings are divergent. Some studies have suggested that resilience plays a partial mediating role in the effect of PA on mental health (41, 42); others have suggested that there is no significant correlation between indicators of PA and mental health after controlling for the variable of resilience (43). Yet, researchers have not used the method of meta-analysis to elucidate the mediating role of resilience and uncover the mechanism by which PA affects mental health. Hence, this gap in the literature emphasizes the need to address this important research void.

### 1.3 Objectives and hypotheses

Considering the lack of consistency in the current research on the relationship between PA and mental health and the possible mediating role of resilience between the two, it is necessary to summarize and analyze the existing findings. Therefore, the primary aim of this study was to assess the strength of the relationship between PA, resilience, and mental health by conducting a meta-analysis of the relevant literature. This analysis would involve constructing a joint correlation matrix among the three variables based on the results of the meta-analysis. Finally, this study aimed to confirm the mediating role of resilience using Structural Equation Modeling. Reviewing the current research, we anticipated a substantial positive correlation between PA and positive indicators of mental health, as well as a significant negative correlation with negative indicators of mental health. We also expected that PA could have a positive impact on positive indicators of mental health and a negative impact on negative indicators of mental health mediated by resilience.

## 2 Materials and methods

### 2.1 Literature retrieval

The systematic review and meta-analysis were conducted in strict adherence to PRISMA guidelines. We searched for the terms sport*/physical activi*/exercis*; resilience/resilient; “life satisfaction”/“positive emotion”/well-being/“positive mood”/mental health/depression/anxiety/“negative emotion”/“negative mood” in databases such as PubMed, Web of Science, ProQuest, Ebsco, China Knowledge, and China Biomedical Database. To identify literature published from its establishment to November 2023. Initially, the following criteria were met: the source of the literature was journal articles, the research content was the relationship between PA and mental health (positive and negative indicators), and the language of the literature was written in Chinese/English. Finally, 3,493 papers were retrieved.

### 2.2 Inclusion and exclusion criteria

**Figure 1:**
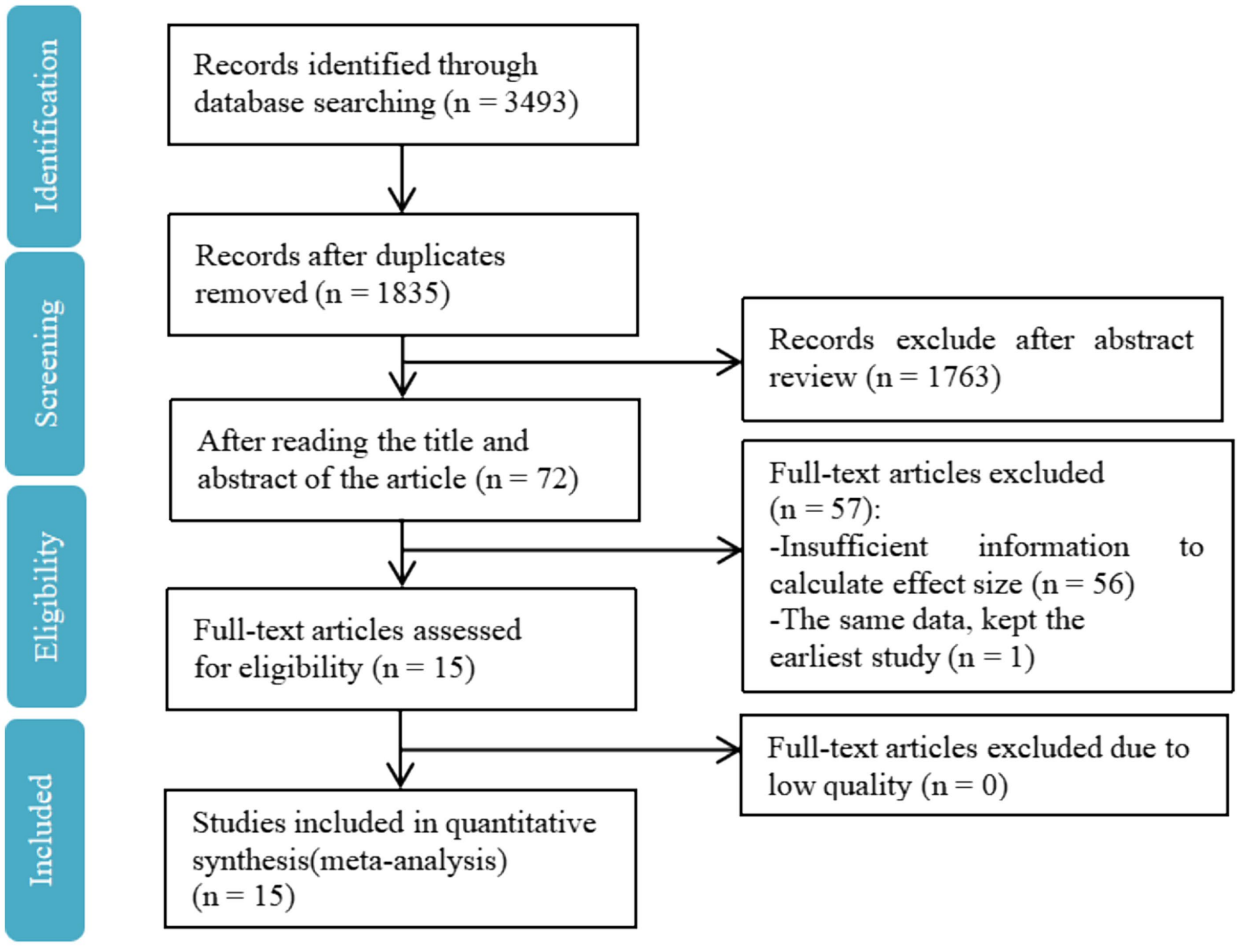
Flow chart of literature screening.

The selection of literature was based on the following criteria: (a) the required studies must be empirical and published (mainly cross-sectional studies, longitudinal studies use baseline data); (b) these studies must cover predictor, mediator, and outcome variables, i.e., PA, resilience, and mental health (which can be measured through any of life satisfaction, subjective well-being, positive affect, depression, anxiety, and negative affect); (c) the sample size and correlation coefficient, or other indicators of transformable data, must be reported; (d) for literature that repeatedly used the same set of data, the earliest one was selected. Figure 1 depicts the literature screening process.

### 2.3 Study coding

The literature data was encoded to incorporate the first author and year, sample size, effect size, and name of the mental health indicator. If the study included multiple samples, the effect sizes were calculated separately for each independent sample. Two researchers finally retrieved the material and conducted the coding separately. In cases of conflicts or inconsistencies, a third researcher was consulted to review and debate the differences and collaboratively resolve the issues. Table 1 depicts a total of 15 papers that were finally included in the present study.

**Table 1:** Basic information on the included meta-analysis studies.

Author, year	Sample size	Mental health indicators	*r*_1_	*r*_2_	*r*_3_	Subjects
Cocozza et al., 2020 (65)	1,182	SWB	0.150	0.240	0.46	Adult
Cui and Zhang, 2022 (66)	1,048	Negative indicator	0.301	−0.357	−0.366	Undergraduate
Ho et al., 2015 (67)	775	Positive indicator	0.250	0.660	0.440	Middle school student
Li et al., 2022 (43)	1,009	Life satisfaction	0.234	0.218	0.618	Elementary school students
Li et al., 2021 (68)	1,214	Negative indicator	0.470	−0.250	−0.330	Undergraduate
Liu, 2020 (41)	1,408	Negative indicator	0.253	−0.357	−0.355	Undergraduate
Wu et al., 2023 (69)	1,248	Positive indicator	0.290	0.210	0.590	Adult
Wu et al., 2022 (70)	402	Positive indicator	0.386	0.180	0.570	Adult
Xin et al., 2023 (71)	451	Anxiety	0.269	−0.301	−0.237	Older adult
Xu et al., 2018a (72)	2,282	Life satisfaction	0.297	0.123	0.254	Middle school student
Xu et al., 2018b (72)	2,282	Negative indicator	0.297	−0.137	−0.258	Middle school student
Yang et al., 2021 (61)	557	SWB	0.36	0.382	0.353	Older people
Zhang et al., 2023 (73)	3,143	SWB	0.249	0.247	0.467	Adolescent
Zhang, 2018 (74)	485	Depression	0.219	−0.315	−0.393	Older adult
Zhang et al., 2022 (75)	1,117	Negative indicator	0.098	−0.087	−0.304	Undergraduate
Zhou and Zhou, 2022 (42)	722	SWB	0.19	0.187	0.341	Undergraduate

*r1 is the correlation coefficient between PA and resilience; r2 is the correlation coefficient between PA and mental health indicators; r3 is the correlation coefficient between resilience and mental health indicators; SWB is subjective well-being.*

### 2.4 Statistical methods

The effect size for the meta-analysis was determined to be the correlation coefficient r. Using Fisher’s *Z* transformation, weighted average effect values were obtained for each group of relationships (44). The interconversion formula is shown below:


$${\mathrm{Fisher}}^{\prime }s\;Z=0.5\times \ln\left(\frac{1+r}{1-r}\right)$$

$$V_{z}=1/\left(n-3\right)$$

$$SE_{z}=\sqrt{V_{z}}$$

$$r=\left(e^{2z}-1\right)/\left(e^{2z}+1\right)$$

**Figure 2:**
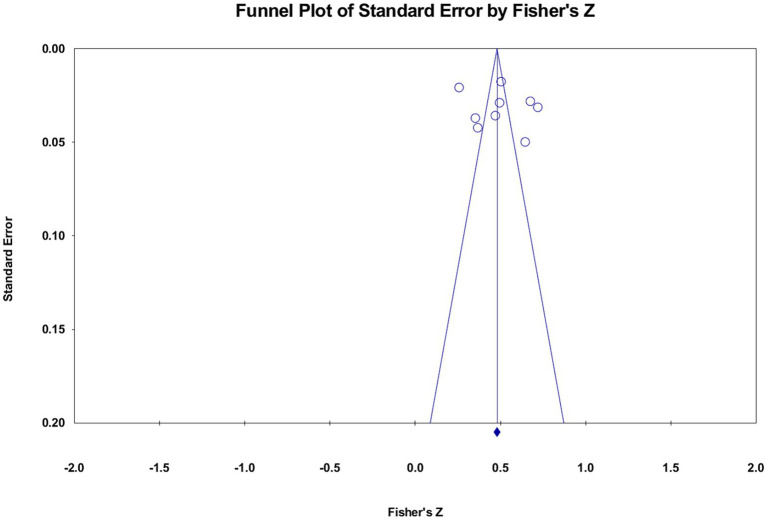
Funnel diagram.

A statistical analysis of effect value merging, heterogeneity test, and publication bias was performed using CMA 3.7. The effect sizes were combined using a random-effects model, and the heterogeneity of the total effect sizes was evaluated using *Q*-values and *I*^2^. Correlation coefficients and confidence intervals of 95% are provided in the study. Publication bias assessments were performed utilizing three methodologies: (a) Fail-safe numbers (Fail-safe *N*) were calculated to determine the number of insignificant studies that would need to be included to render the total effect value statistically insignificant (45). (b) A funnel plot was created to evaluate the existence of publication bias by visually analyzing the symmetry of the plot. If the effect values are predominantly clustered in the top middle of the funnel and exhibit symmetry, it suggests the absence of publication bias, as depicted in Figure 2. (c) If both the approaches above confirmed the presence of publication bias, the funnel plot was rectified using the trim-and-fill method to derive an adjusted effect size (46).

Based on meta-analytic structural equation modeling (MASEM) theory, the path analysis and the mediating effects of resilience were validated by the great likelihood method using the WebMASEM application^1^ (47).

## 3 Results

Fifteen articles containing 17,043 subjects were included in this study. Among them, 10 articles were studied in the adolescent and college student population; three were adults, and two were older adults.

### 3.1 Heterogeneity test

As depicted in Table 2, it was found that after testing, the *I*^2^ values of all groups exceeded 70%, a high degree of heterogeneity in the included studies (*p* < 0.01), so it was more accurate to use a random effects model to combine the effect sizes.

**Table 2:** Results of meta-analysis.

Variable relationship	*k*	*r*	95% Confidence interval		*z*	*p*	*Q*	df	*p*	*I*^2^	Fail-safe *N*
			Lower limit	Up limit							
PA-PI	8	0.288	0.166	0.402	4.496	0.000	283.162	7	0.000	97.53%	1,149
Re-PI	8	0.463	0.362	0.552	8.082	0.000	249.462	7	0.000	97.19%	4,517
PA-NI	7	−0.259	−0.342	−0.171	−21.621	0.000	99.465	6	0.000	93.97%	875
Re-NI	7	−0.321	−0.360	−0.280	−29.069	0.000	22.667	6	0.001	73.53%	1,480

PA is PA; Re is resilience; PI is positive indicators of mental health; NI is negative indicators of mental health; *k* is the number of studies; *r* is the effect size.

### 3.2 Publication bias test

A visual evaluation using a funnel plot indicated that most of the effect values for each set of associations were clustered in the central top region of the funnel and were evenly distributed on both sides of the overall effect size. The fail-safe *N* measure was calculated (Table 2), which yielded a critical value greater than 5*k* + 10 for each item. Furthermore, no other studies were identified for inclusion during the trim-and-fill process. Therefore, there was no publication bias in the current meta-analysis.

### 3.3 Main effects test

The mean weighted correlation coefficients between PA and positive indicators of mental health were 0.288 (95% CI, 0.166–0.402). Similarly, the average weighted correlation coefficients between resilience and positive indicators of mental health were 0.463 (95% CI, 0.362–0.552). Conversely, the average weighted correlation coefficients between PA and negative indicators of mental health were −0.259 (95% CI, −0.342 to −0.171), and between resilience and negative indicators of mental health were −0.321 (95% CI, −0.360 to −0.280). The results of the *z*-value and *p*-value indicated that these effect sizes were statistically significant. Based on Cohen’s criteria (48), a value of ∣*r*∣ ≤ 0.10 is considered a small effect, ∣*r*∣ = 0.30 is considered a medium effect, and ∣*r*∣ ≥ 0.50 is considered a large effect. This suggests that there is a medium to high positive correlation between PA and resilience with positive indicators of mental health and a medium to high negative correlation with negative indicators of mental health.

### 3.4 Mediation effect test

The mediation effects of resilience were tested using the MASEM theory. Table 3 presents the joint correlation matrix, which was calculated using the Web MASEM application to obtain the results.

**Table 3:** Physical activity, resilience, and mental health correlation matrix.

Variable	PA_1_	Re_1_	PA_2_	Re_2_
Re_1_	0.263	1		
PI	0.27	0.453		
Re_2_			0.273	1
NI			−0.256	−0.319

PA_1_ is a PA that impacts positive indicators of mental health; Re_1_ is resilience that impacts positive indicators of mental health; PA_2_ is a PA that impacts negative indicators of mental health; Re_2_ is resilience that impacts negative indicators of mental health; PI is positive indicators of mental health; NI is negative indicators of mental health.

Second, the lavaan syntax^2^ was composed. Path analyses and mediation effects tests were performed using the WebMASEM program. The fit indices of SEM showed that the model was saturated (*χ*^2^ < 0.001, df = 0, CFI = 1.00, TLI = 1.00, RMSEA = 0), indicating that the model fits well with the data.

Table 4 depicts the results of the test. Both PA and resilience can directly predict positive mental health indicators, with path coefficients of 0.161 (95% CI: 0.040–0.283) and 0.411 (95% CI: 0.316–0.506), respectively. PA can indirectly impact positive indicators of mental health by influencing resilience. The indirect effect value is 0.108 (95% CI: 0.080–0.141), allowing for 40.15% of the total effect value. PA and resilience had a direct negative impact on mental health, with path coefficients of −0.182 (95% CI: −0.0275 to −0.089) and −0.270 (95% CI: −0.319 to −0.221), respectively. Furthermore, PA indirectly affected negative indicators of mental health through the mediating effect of resilience, with an indirect effect value of −0.074 (95% CI: −0.100 to −0.051), accounting for 28.91% of the total effect value. The 95% confidence intervals for all effect sizes did not contain 0. Thus, the mediating role of resilience in the relationship between PA and mental health was established.

**Table 4:** Path analysis of the mediating effect of resilience.

Mediating variable	*k*	*a*	CI*_a_*	*b*	CI*_b_*	*ab*	CI*_ab_*	*c*	CI*_c_*	*d*
PA-Re-PI	8	0.263	0.216, 0.313	0.411	0.316, 0.506	0.108	0.080, 0.141	0.161	0.040, 0.283	0.269
PA-Re-NI	7	0.273	0.183, 0.361	−0.270	−0.319, −0.221	−0.074	−0.100, −0.051	−0.182	−0.275, −0.089	−0.256

*k* is the number of studies; PA is PA; Re is resilience; PI is positive indicators of mental health; NI is negative indicators of mental health; *a* is the effect of the predictor variable (PA) on the mediator variable (Re); *b* is the effect of the mediator variable (Re) on the outcome variables (PI, NI); *ab*: the indirect effect of the predictor variable on the outcome variable through the mediator variable; *c*: the direct effect of the predictor variable on the outcome variable; *d*: the total effect of the predictor variable on the outcome variable.

## 4 Discussion

PA has an important place in people’s lives. Overall, engaging in PA can alleviate stress, regulate negative emotions, enhance life satisfaction, and foster mental well-being (49, 50). From a physiological perspective, engaging in PA leads to increased production and release of endorphins, decreased adrenaline levels, and stimulated logical thinking and reasoning abilities. Consequently, the most immediate outcome of PA is the experience of pleasure and a sense of well-being (51, 52). This is also indicated by our findings that PA is correlated with positive indicators of mental health and that PA can positively influence positive indicators of mental health. Increasing happy emotions during exercise can alleviate the impact of negative emotions and mitigate depression, anxiety, and work-related stress. Moreover, PA can play a crucial role in treating anxiety and depression by improving various physiological factors (53), as illustrated by the results of the present study, where PA showed a negative correlation with negative indicators of mental health. Nevertheless, researchers have already emphasized that the relationship between PA and mental health differs considerably across different life domains (27); moreover, certain specific groups, such as older adults (54) and smokers (55), may experience negative emotions due to the physical discomfort or risks associated with exercise. Hence, it is imperative to establish a scientific exercise prescription to implement exercise interventions for mental health issues. The advent of an information-driven and sophisticated society has resulted in a decline in the level of PA among people, which inevitably leads to mental health issues. It has been suggested that physical inactivity is the leading cause of death globally, which has a huge impact on the prevalence of non-communicable diseases such as mental health issues (56). Therefore, the impact of physical inactivity on mental health remains a concern for the future.

Resilience, an inherent quality or skill to deal with challenges, is closely linked to mental well-being. Individuals with a high level of resilience tend to have a more positive outlook on cognitive matters. The results of our study indicated a significant positive association between resilience and favorable mental health indicators. Moreover, resilience has a significant negative correlation with negative indicators. Researchers presented a comprehensive meta-analysis (28) that thoroughly clarifies the connection between resilience and mental health. Those who possess resilience are more capable of confronting challenges, positively dealing with them, seeking support, and effectively solving problems (57). This resilience acts as a protective shield, safeguarding individuals from the negative emotional effects of unfavorable occurrences (58). The findings of the present study indicate that the correlation between resilience and mental health is notably stronger than that of physical exercise. This could be attributed to the fact that resilience has a more immediate impact on mental health than PA (42). Furthermore, resilience, as a characteristic of one’s personality, exhibits greater stability and a more robust association with an individual’s mental well-being than physical exercise, which is an external protective factor. This trait of resilience suggests that it may mediate other distal variables affecting mental health. Therefore, this needs to be focused on in studying mental health issues.

Physical activity was a significant predictor of resilience, consistent with previous reports (59). Individual resilience is not static and can be influenced by both positive and negative factors (60). PA as a protective factor, PA can enhance the development of individual resilience (61). Research has shown that regular participation in PA can effectively reduce physiological stress levels, foster emotional balance, strengthen self-control, and boost mental well-being, thereby leading to increased levels of resilience (62). Moreover, resilience can be influenced by various factors, such as the type and intensity of activity and personal beliefs and habits (63). There are many intricate relationships between PA and resilience and mental health (30). This study is the first to investigate how resilience mediates PA and mental health using a meta-analytic structural equation model (MASEM). The results showed that PA not only directly affects mental health but also positively predicts positive indicators of mental health and negatively predicts negative indicators of mental health through the mediating role of resilience. Even after the introduction of resilience, the direct effect of PA and mental health was still significant, indicating that resilience plays a partially mediating role, with the mediating effects accounting for 40 and 29%, respectively. Our study revealed that resilience had a stronger mediating influence on positive mental health measures than negative ones. This implies that positive mental health outcomes may favor a person’s trait resilience (28) and can further increase the weight of resilience in the system of PA-mental health relationships. Resilience shields individuals from the consequences of difficult circumstances by following four primary potential routes: diminishing the likelihood of being affected, mitigating adverse ripple effects, bolstering psychosocial abilities like self-esteem and self-efficacy, and providing chances for adaptive responses (64). Therefore, consistently engaging in PA can indirectly improve mental health by bolstering psychological resilience and boosting an individual’s self-esteem and self-efficacy.

## 5 Limitations and future directions

In order to validate the mediating effect of resilience, it was necessary to include three variable indicators: PA, mental health, and resilience. Nevertheless, this reduced the sample size, which may have affected the accuracy of the effect values associated with PA and mental health.

The included studies had significant variation and were restricted in number. This study did not examine the moderating effects of characteristics such as subject age, gender, and type of measurement instrument. Therefore, further research is required in the future.

From the perspective of the research subjects included in the study, there are very few relevant studies on older adults. As China, a densely populated nation, experiences an aging population, it becomes imperative to allocate more focus and study toward the mental well-being of the older adults.

China has been the fastest-growing country in the world in the past two decades. The lack of physical activity of people caused by informatization and intelligence has become a major cause of non-communicable diseases, including mental health problems. Hence, there is an urgent need to focus on studies related to physical inactivity in the future.

## 6 Conclusion

This study was the first to explore the relationship between PA, resilience, and mental health using the MASEM approach. Our findings suggested that PA is positively correlated with positive indicators of mental health and negatively correlated with negative indicators of mental health. Moreover, PA can positively influence mental health through the mediating role of resilience.

## Data Availability

The original contributions presented in the study are included in the article/supplementary material, further inquiries can be directed to the corresponding author.

## Footnotes

^1^ https://sjak.shinyapps.io/webMASEM/

^2^Using the positive indicators of mental health as an example: the direct effects are # Regression coefficients; Re ~ b21*PA; PI ~ b31*PA + b32*Re; # Variances; PA ~~ 1*PA; Re ~~ p22*Re; PI ~~ p33*PI; and the indirect effects are # Regression coefficients; Re ~ beta1*PA; PI ~ beta2*Re + PA; # Variances; PA ~~ 1*PA; Re ~~ Re; PI ~~ PI.
