# Supplementary material for: The mediating effect of resilience between physical activity and mental health: a meta-analytic structural equation modeling approach
Source: Front Public Health. 2024 Oct 1;12:1434624. doi: 10.3389/fpubh.2024.1434624 (PMC11473373; doi:10.3389/fpubh.2024.1434624)
Supplement: Supplementary file 1 [file Data_Sheet_1.docx]

Supplementary Material

The mediating effect of resilience between physical activity and mental health: a meta-analytic structural equation modeling approach

Hao Lin^1*^, Yuying Zhu^1^, Qingzao Liu^1,2^ Shan Li^1^

*** Correspondence:**

linhao@cdu.edu.cn

# Search strategies

## English search strategy(Take the Web of Science for example)

Set#1: TS=(mental health) OR TS=(well-being) OR TS=("life satisfaction") OR TS=("positive emotion") OR TS=("positive mood") OR TS=(depression) OR TS=(anxiety) OR TS=("negative emotion") OR TS=("negative mood")

Set#2: TS=(exercis*) OR TS=(physical activit*) OR TS=(sport*)

Set#3: TS=(resilience OR resilient)

Set#4: (#1 AND #2 AND #3)

All sets:

Indexes = SCI-EXPANDED, SSCI, CPCI-S, CPCI-SSH.

Timespan= Establishment-2023.11

Language=English

## Chinese search strategy(CNKI)

Set#1: SU=(心理健康 OR 幸福感 OR 生活满意度 OR 情绪 OR 抑郁 OR 焦虑)

Set#2: SU=(运动 OR 体育活动 OR 身体活动 OR 体育锻炼 OR 体力活动)

Set#3: SU=(心理韧性 OR 心理弹性)

Set#4: (#1 AND #2 AND #3)

All sets:

Indexes = 北大核心, CSSCI, CSCD

Timespan= Establishment -2023.11
